# Ruthenium(II) Polypyridyl Complexes Containing Simple Dioxo Ligands: a Structure‐Activity Relationship Study Shows the Importance of the Charge

**DOI:** 10.1002/cbic.202200398

**Published:** 2022-08-26

**Authors:** Albert Gandioso, Alessio Vidal, Pierre Burckel, Gilles Gasser, Enzo Alessio

**Affiliations:** ^1^ Department of Chemical and Pharmaceutical Sciences University of Trieste 34127 Trieste Italy; ^2^ Chimie ParisTech PSL University CNRS Institute of Chemistry for Life and Health Sciences Laboratory for Inorganic Chemical Biology 75005 Paris France; ^3^ Université de Paris Institut de physique du globe de Paris CNRS 75005 Paris France; ^4^ Chimie ParisTech PSL University CNRS Institute of Chemistry for Life and Health Sciences Laboratory for Inorganic Chemical Biology, F- 75005 Paris France

**Keywords:** bioinorganic chemistry, cancer, medicinal inorganic chemistry, polypyridyl complexes, ruthenium

## Abstract

Cancer is one of the main causes of death worldwide. Platinum complexes (i. e., cisplatin, carboplatin, and others) are currently heavily used for the treatment of different types of cancer, but unwanted effects occur. Ruthenium complexes have been shown to be potential promising alternatives to these metal‐based drugs. In this work, we performed a structure‐activity relationship (SAR) study on two small series of Ru(II) polypyridyl complexes of the type [Ru(L1)_2_(O^O)]Cl_n_ (**3**–**8**), where L1 is 4,7‐diphenyl‐1,10‐phenantroline (DIP) or 1,10‐phenantroline (phen), and O^O is a symmetrical anionic dioxo ligand: oxalate (ox, n=0), malonate (mal, n=0), or acetylacetonate (acac, n=1). These two self‐consistent series of compounds allowed us to perform a systematic investigation for establishing how the nature of the ligands and the charge affect the anticancer properties of the complexes. Cytotoxicity tests on different cell lines demonstrated that some of the six compounds **3**–**8** have a promising anticancer activity. More specifically, the cationic complex [Ru(DIP)_2_(η^2^‐acac)]Cl (**4**) has IC_50_ values in the mid‐nanomolar concentration range, lower than those of cisplatin on the same cell lines. Interestingly, [Ru(DIP)_2_(η^2^‐acac)]Cl was found to localize mainly in the mitochondria, whereas a smaller fraction was detected in the nucleus. Overall, our SAR investigation demonstrates the importance of combining the positive charge of the complex with the highly lipophilic diimine ligand DIP.

## Graphical abstract

**A structure‐activity relationship** study on two self‐consistent series of Ru(II) polypyridyl complexes with anionic dioxo ligands showed the importance of combining the positive charge of the complex with a highly lipophilic diimine ligand for obtaining good cellular uptake and cytotoxicity.

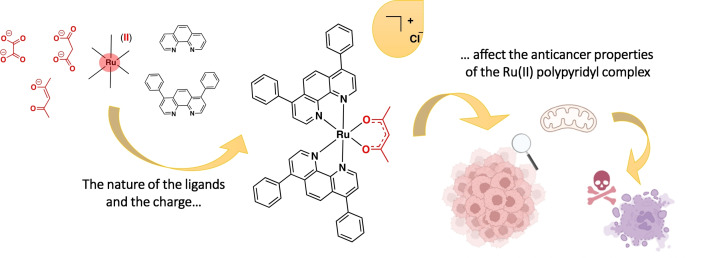

## Introduction

Inert Ru(II) polypyridyl complexes hold tremendous potential as chemotherapeutic agents against cancer, both in the presence of visible light – i. e., as photosensitizers for photodynamic therapy (PDT) – and in its absence.[^1^, ^2^, ^3^, ^4^, ^5^, ^6^] The spearhead among metal complexes investigated as potential PDT agents is TLD‐1433, a bis‐heteroleptic Ru(II) polypyridyl complex that is in phase II clinical trial against high‐risk non‐muscle invasive bladder cancer (NMIBC),^[7]^ and is being also investigated against other cancer types.^[8]^ On the other hand, Ru(II) polypyridyl complexes have been studied for several years as cytotoxic agents for anticancer therapy.[^9^, ^10^, ^11^, ^12^, ^13^, ^14^, ^15^, ^16^, ^17^, ^18^, ^19^, ^20^, ^21^, ^22^] In addition to exerting cytotoxic activity, such compounds were found to affect various cell properties crucial for metastasis formation and development, such as detachment, motility, and invasion.[^19^, ^20^, ^21^, ^22^]

**Figure 1:**
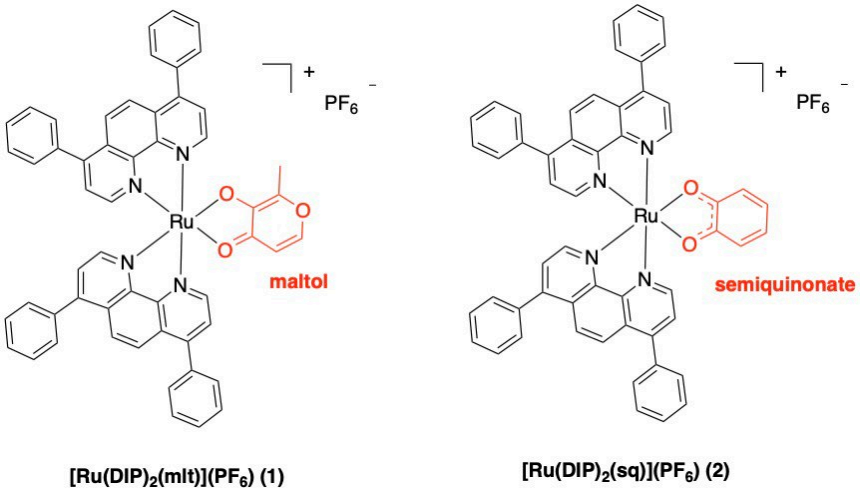
Two representative examples of Ru(II)‐dioxo polypyridyl complexes investigated as potential anticancer drugs by our groups.[^33^, ^34^, ^35^]

Among the plethora of Ru(II) polypyridyl complexes, those bearing one dioxo ligand (O^O) have shown particular promise as potential anticancer agents.[^23^, ^24^, ^25^, ^26^, ^27^, ^28^, ^29^, ^30^, ^31^, ^32^] Within this context, we recently reported that [Ru(DIP)_2_(mlt)](PF_6_) (**1**) (Figure 1, where DIP is 4,7‐diphenyl‐1,10‐phenantroline, and mlt is deprotonated maltol, a flavour‐enhancing agent approved by the FDA) has higher activity than cisplatin against different cell lines in 2D model and on HeLa MultiCellular Tumour Spheroids (MCTS).^[33]^ The complex was found to bind to Human Serum Albumin (HSA) via intermolecular interactions and to be efficiently internalized by HeLa cells through a passive transport mechanism accumulating mainly in the nucleus and mitochondria. Compound **1** is structurally similar to the complex [Ru(DIP)_2_(sq)](PF_6_) (**2**) (Figure 1, sq=semiquinonate), previously investigated by Gasser and co‐workers, which also was found to have cytotoxicity in the nanomolar concentration range in several cancer cell lines (i. e., higher than cisplatin), and a very promising *in vivo* activity.^[34]^ A small series of similar complexes, resulting from the coordination of substituted catecholate‐type dioxo ligands to the same {Ru(DIP)_2_} core, was investigated as well.^[35]^ All compounds were isolated and studied as racemic mixtures of Δ and Λ enantiomers.

**Figure 2:**
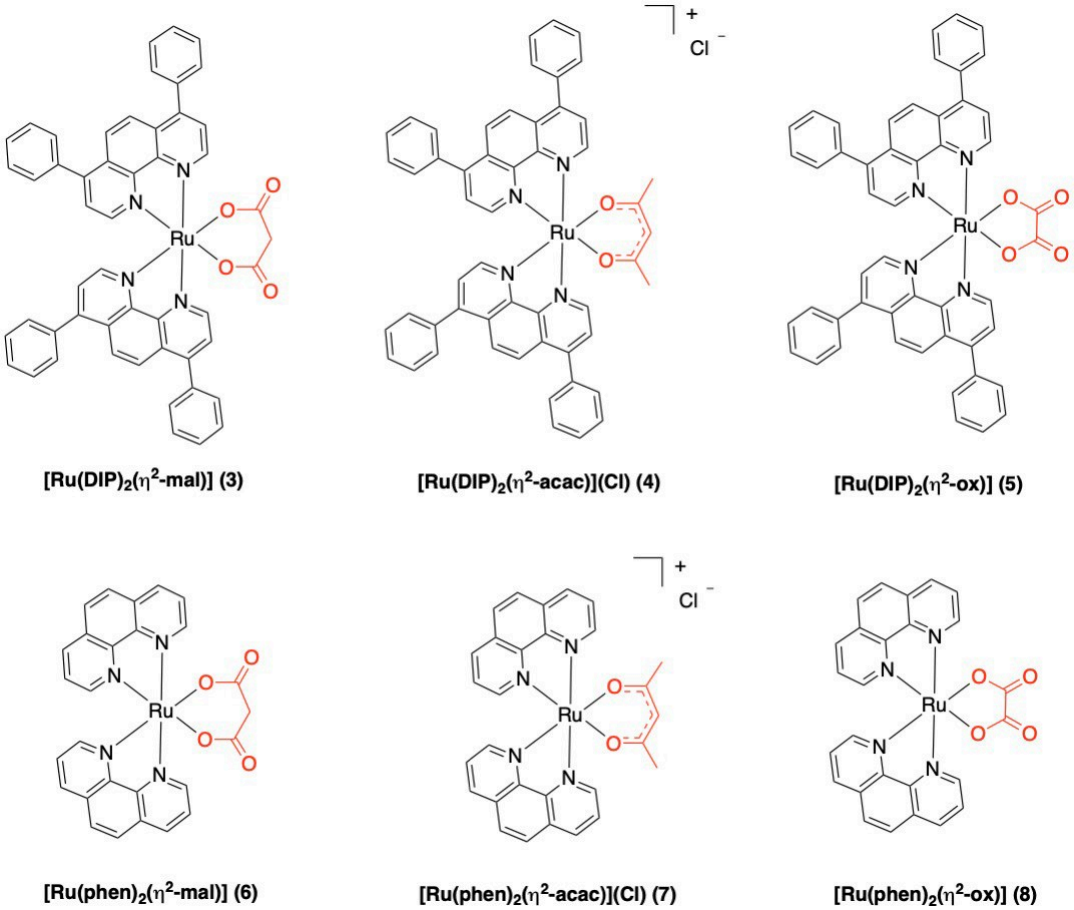
Schematic structures of the ruthenium(II) complexes **3**–**8**.

We recently developed a synthetic strategy for the microwave‐assisted efficient preparation of bis‐heteroleptic Ru(II) polypyridyl compounds [Ru(L1)_2_(L2)]^2+^ that involves the isolation of intermediates of the type [Ru(L1)_2_(O^O)]Cl_n_, where O^O is a symmetrical anionic dioxo ligand.^[36]^ The dioxo complexes are neutral (n=0) when O^O is oxalate (ox) or malonate (mal), mono‐cationic (n=1) when O^O is acetylacetonate (acac). Having this synthetic capability at hand, we decided to extend the studies mentioned above and investigated how the nature and charge of the dioxo ligand affected the anticancer properties of the {Ru(DIP)_2_} core. For this purpose, we prepared a small series of complexes, both neutral and cationic: [Ru(DIP)_2_(η^2^‐mal)] (**3**), [Ru(DIP)_2_(η^2^‐acac)]Cl (**4**), and [Ru(DIP)_2_(η^2^‐ox)] (**5**) (Figure 2). In addition, with the aim of assessing the relevance of the diimine ligand, the corresponding complexes bearing 1,10‐phenanthroline (phen) in place of DIP were prepared and investigated as well: [Ru(phen)_2_(η^2^‐mal)] (**6**), [Ru(phen)_2_(η^2^‐acac)]Cl (**7**), and [Ru(phen)_2_(η^2^‐ox)] (**8**) (Figure 2). The cytotoxicity of compounds **3**–**8** was evaluated in monolayer cultures of the CT‐26 (mouse colon adenocarcinoma), PC‐3 (human Caucasian prostate adenocarcinoma) cell lines, and ‐ for comparison ‐ on the non‐tumorigenic RPE‐1 (human normal retina pigmented epithelial) cell line. Cisplatin and [Ru(DIP)_2_(mlt)](PF_6_) (**1**) were tested in the same cell lines as positive and additional controls. In addition, the partition coefficient measurement and the cellular uptake for the complexes **3–8** were measured. Finally, we investigated the mechanism of action of complexes **3**–**8**, and their influence on the cellular metabolism of CT‐26 cells through the Seahorse Bioanalyzer.

## Results and Discussion

### Synthesis of the ruthenium(II) polypyridyl dioxo complexes

**Figure 3:**
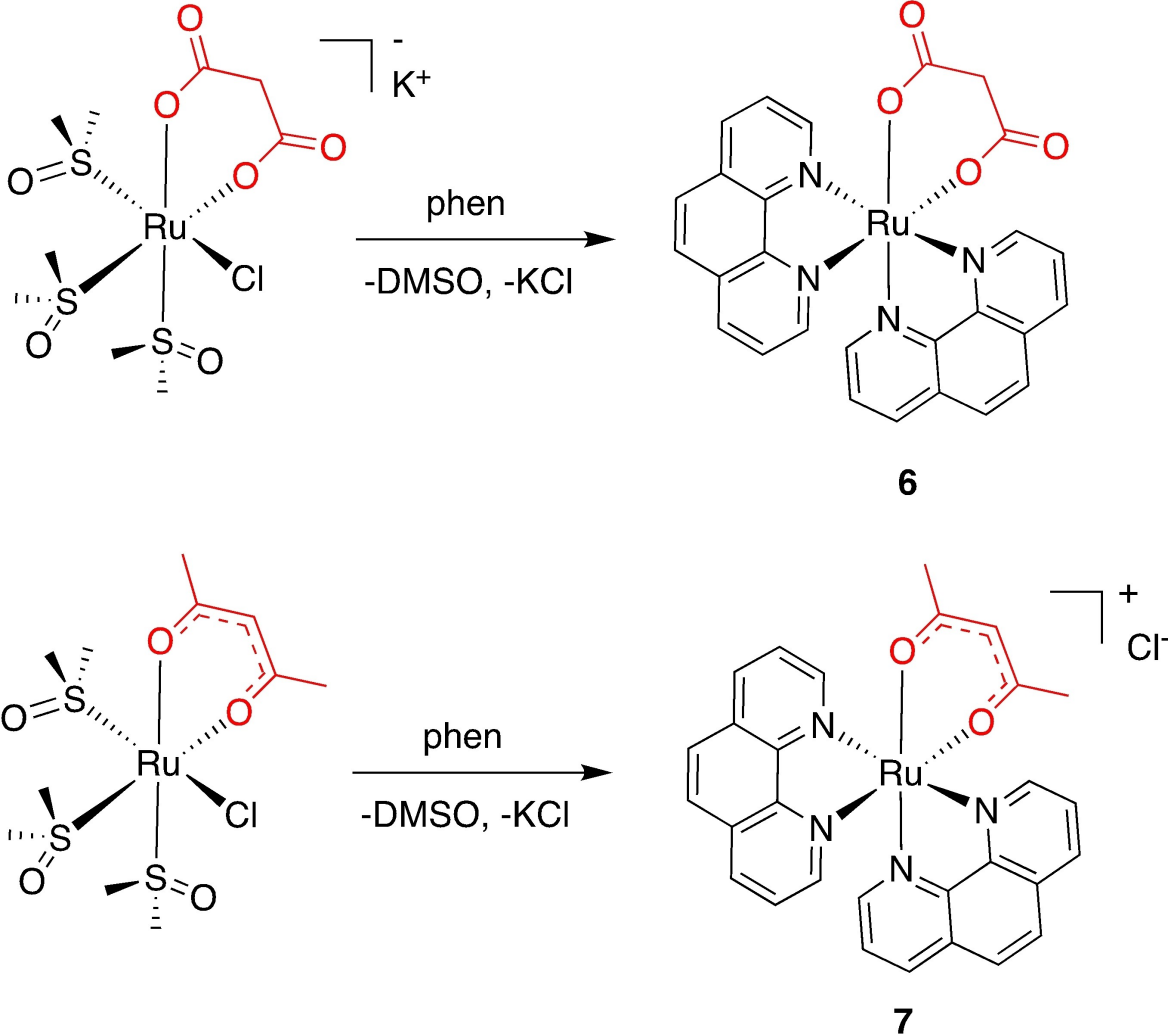
General procedure for the preparation of the complexes **3**–**8** exemplified for a neutral (**6**) and a cationic (**7**) derivative.

As mentioned above, we recently described how *cis*‐locked Ru(II)‐DMSO precursors with dioxo ligands can be exploited for the efficient microwave‐assisted synthesis of bis‐heteroleptic polypyridyl compounds.^[36]^ Ruthenium(II) complexes **3**–**8** were obtained as intermediates following this novel approach, as exemplified in Figure 3 for a neutral (**6**) and a cationic (**7**) complex. Compounds **3**–**8** are all soluble in DMSO, chloroform, and in aqueous media containing 1 % of DMSO.

### Stability studies in DMSO, medium cell culture, and human plasma

The biological activity of anticancer drugs candidates is strongly influenced by their stability in DMSO, medium cell culture, and human plasma. For this reason, we performed stability studies of complexes **3**–**8** at 37 °C in DMSO and DMEM medium (Gibco) (Supporting Information Figures S1 ‐ S2). Importantly, no significant changes in the absorption spectra in DMSO and medium cell culture after 24 h were observed for the series of DIP complexes **3**–**5**. In contrast, a small decrease in the absorption band intensity, more marked for the neutral complexes, was observed in the spectra of the phen compounds **6**–**8**, probably due to slow partial precipitation. Finally, the stability of the compounds was investigated by incubating them in human plasma at 37 °C for 48 h, followed by UV‐Vis and HPLC analysis. The UV‐vis spectra and HPLC chromatograms for compounds **3**–**5**, **7**, and **8** (Supporting Information Figures S3–S5) were very similar before and after incubation in human plasma, indicative of good stability of the complexes under biological conditions. A change in the UV spectrum's shape, indicating chemical transformation, was observed only for the malonate complex **6** (similar to what observed in DMEM, Figure S2). After 48 h, only 15 % of the complex was still intact, as observed by HPLC (Supporting Information Figure S5).

### Cytotoxicity studies on 2D monolayer cells

The first step toward the biological investigation of complexes **3**–**8** was the evaluation of cell viability in monolayer cultures of CT‐26 (mouse colon adenocarcinoma), PC‐3 (human Caucasian prostate adenocarcinoma), and RPE‐1 (human normal retina pigmented epithelial) cell lines using a fluorometric cell viability assay with the Resazurin reagent. Cisplatin and **1** were tested in the same cell lines as positive and additional controls. The IC_50_ values of the tested compound are reported in Table 1.

**Table 1:** IC_50_ [μM] values for cisplatin, [Ru(DIP)_2_(mlt)](PF_6_) (**1**), [Ru(DIP)_2_(η^2^‐mal)] (**3**), [Ru(DIP)^2^(η^2^‐acac)]Cl (**4**), [Ru(DIP)_2_(η^2^‐ox)] (**5**), [Ru(phen)_2_(η^2^‐mal)] (**6**), [Ru(phen)_2_(η^2^‐acac)]Cl (**7**), and [Ru(phen)_2_(η^2^‐ox)] (**8**) in three different cell lines (48 h).

	CT‐26	PC‐3	RPE‐1
cisplatin	1.94±0.72	10.32±1.14	20.13±3.91
[Ru(DIP)_2_(mlt)](PF_6_)	0.37±0.12	1.84±0.23	0.67±0.14
**3**	5.63±0.34	4.92±0.33	5.00±0.70
**4**	0.41±0.10	0.62±0.19	0.50±0.12
**5**	24.12±2.50	21.05±2.29	14.50±3.00
**6**	>100	>100	>100
**7**	10.30±0.74	>100	>100
**8**	>100	>100	>100

Compounds **3**–**5** with the DIP ligand present the highest cytotoxicity with IC_50_ values in the micromolar range. In contrast, phen compounds **6**–**8** have much lower cytotoxicity with IC_50_ higher than 100 μM (except for **7** in the CT‐26 cell line). Of particular interest, in the three cell lines the cationic compound **4** exhibits IC_50_ values in the mid‐nanomolar concentration range, ca. one order of magnitude lower than those of the corresponding neutral complexes. The IC_50_ values of **4** are comparable with those of **1** in the same cell lines,^[33]^ as well as with those of other complexes previously tested by us.[^34^, ^35^] The results also highlight the importance of the DIP ligand instead of phenanthroline for observing cytotoxicity. This is probably due to the higher lipophilicity of this ligand, allowing a better cellular uptake (see below). Disappointingly, this series of novel complexes – similarly to **1** ‐ did not display any selectivity towards the CT‐26 and PC‐3 cancer cell lines compared to the healthy cell line RPE‐1. However, overall, complex **4** is still the best candidate among the complexes investigated in the 2D model.

### ICP‐MS cellular uptake studies

The cellular uptake of the complexes **3**–**8** was then investigated in CT‐26 cells by determining the amount of Ru inside the cells using inductively coupled plasma mass spectrometry (ICP‐MS) after 2 h of incubation at 5 μM. The cationic complex [Ru(DIP)_2_(η^2^‐acac)]Cl (**4**) was found to have the most significant uptake, almost three times larger compared to the phen counterpart [Ru(phen)_2_(η^2^‐acac)]Cl (**7**). This is most probably due to the higher lipophilicity of the DIP ligand compared to phen. The uptake of **4** is also ca. three times larger than that of the similar DIP reference complex **1** (in this case, however, the comparison might be affected by the different counter‐ion, Cl vs PF_6_). In contrast, the neutral complexes with the DIP ligand (**3** and **5**) present a lower uptake (ca. 50 %) compared the phen analogues **6** and **8**. This observation is rather counter‐intuitive since compounds **3** and **5** have a higher cytotoxicity than **6** and **8**. The lower uptake of the DIP complexes **3** and **5** might be due to the lower solubility of these complexes in the cell culture medium. However, taken together, the uptake and cytotoxicity data imply that **3** and **5** have a higher specific cytotoxic activity compared to the phen analogues **6** and **8**, reasonably attributable to their higher lipophilicity.

**Figure 4:**
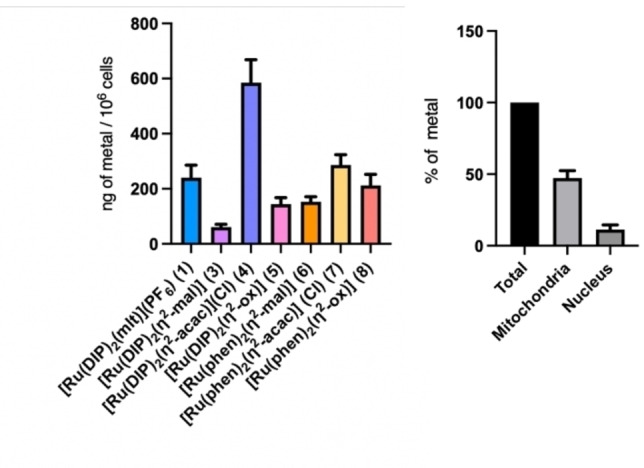
Left: Cellular uptake of compounds **3**–**8** and of the reference complex [Ru(DIP)_2_(mlt)](PF_6_) (**1**) in CT‐26 cells. Right: Intracellular distribution of [Ru(DIP)_2_(η^2^‐acac)]Cl (**4**).

Additionally, we choose the best candidate from the cytotoxicity experiments, namely [Ru(DIP)_2_(η^2^‐acac)]Cl (**4**), to explore its cellular localisation. For this, we performed cellular fractionation experiments to ascertain in which organelle it localizes. The relative biodistribution of **4** among the different subcellular compartments is presented in Figure 4 (right). The complex was found to localize mainly in the mitochondria (ca. 50 % of the total amount of Ru), whereas a smaller fraction (ca. 10 %) was detected in the nucleus. Interestingly, the similar cationic reference compound **1**, contrary to complex **4**, was found to accumulate more in the nucleus than in mitochondria.^[33]^

### Log P value determination

**Figure 5:**
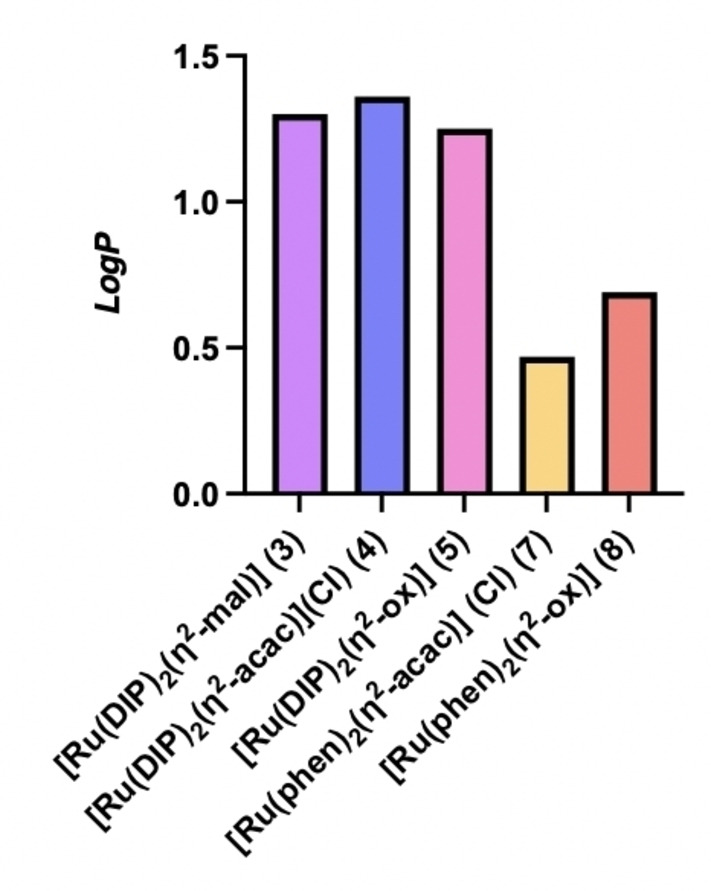
Octanol/PBS partition coefficients of complexes **3**–**5**, **7**, and **8**.

The lipophilicity and hydrophilicity of the compounds was determined by measuring their *n*‐octanol/PBS partition coefficient (log P) by the shake‐flask method. As shown in Figure 5, all compounds were lipophilic, with a preferential partition in the octanol layer. Specifically, as expected, the DIP complexes **3**–**5** present values of lipophilicity higher (ca. double) than the phen complexes **7** and **8** (due to its poor stability, the log P of the complex **6** could not be precisely measured using this method).

### Metabolic studies and Mito stress test

**Figure 6:**
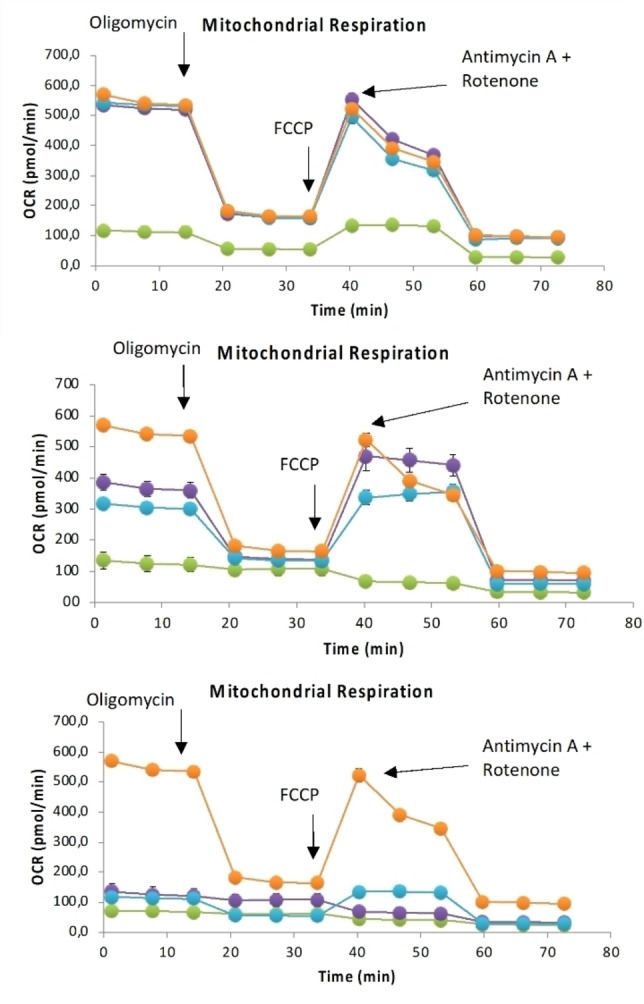
Mito Stress Test profile in CT‐26 cells after 24 h treatment; oxygen consumption rate changes after treatment with specific electron transport chain inhibitors, namely, oligomycin (inhibitor of ATP synthase (complex V)), FCCP (uncoupling agent), antimycin‐A (complex III inhibitor), and rotenone (complex I inhibitor). Top: The DIP series of complexes [Ru(DIP)_2_(η^2^‐mal)] (**3**, •), [Ru(DIP)_2_(η^2^‐acac)]Cl (**4**, •), and [Ru(DIP)_2_(η^2^‐ox)] (**5**, •). Middle: The phen series of complexes [Ru(phen)_2_(η^2^‐mal)] (**6**, •), [Ru(phen)_2_(η^2^‐acac)]Cl (**7**, •), and [Ru(phen)_2_(η^2^‐ox)] (**8**, •). Bottom: Comparison between the cationic complexes [Ru(DIP)_2_(mlt)](PF_6_) (•), [Ru(DIP)_2_(η^2^‐acac)]Cl (**4**, •), and [Ru(phen)_2_(η^2^‐acac)]Cl (**7**, •). In all graphs the orange dots represent untreated cells.

To further study the mechanism of action of complexes **3**–**8**, their influence on the cellular metabolism of CT‐26 cells was investigated. Figure 6 represents the Mito stress test profile after 24 h of treatment; the oxygen consumption rate (OCR) changes with specific electron‐transport chain inhibitors. Oligomycin (inhibitor of ATP synthase (complex V)), FCCP (uncoupling agent), antimycin‐A (complex III inhibitors), and rotenone (complex I inhibitor) were employed in this study. In general, both cationic compounds **4** and **7** affected the mitochondrial processes. It is evident from the low basal respiration and the inhibition of ATP production compared to untreated cells (orange dots) that the mitochondrial membrane of the cells treated with such complexes lost the capacity to restore the proton balance when treated with an uncoupling agent (FCCP). The maximal respiration (the OCR value when the mitochondrial membrane is uncoupled) and spare respiratory capacity (difference in the OCR values between maximal respiration and basal respiration) of the cells were reduced compared to those of untreated cells (Figure 6). The low basal respiration observed in cells treated with the cationic complexes **4**, **7**, and **1** in comparison to untreated cells indicates a severe impairment of mitochondrial respiration. The results with complex **4** are consistent with its measured large mitochondrial uptake (see above). Interestingly, the effect of **1** is similar to that of **4**, even though it has a lower mitochondrial uptake.^[33]^

Conversely, the neutral DIP and phen compounds show similar profiles as the untreated cells, suggesting that they enter into the mitochondria to a lesser extent (if at all). As a consequence, the cytotoxic activity of the neutral DIP complexes **3** and **5** might have a different origin compared to that of the cationic complex **4**.

## Conclusions

In summary, we performed a structure‐activity relationship (SAR) study based on the promising activity expressed by the maltol (mlt) complex [Ru(DIP)_2_(mlt)](PF_6_) (**1**) that our groups recently reported.^[33]^ We developed two self‐consistent small series of Ru(II) polypyridyl complexes of the type [Ru(L1)_2_(O^O)]Cl_n_, where O^O is a symmetrical anionic dioxo ligand (**3** ‐ **8**). They allowed us to investigate how the nature of the diimine ligand (L1=1,10‐phenanthroline (phen) or 4,7‐diphenyl‐1,10‐phenanthroline (DIP)), of the dioxo ligand (O^O=oxalate (ox), malonate (mal), acetylacetonate (acac)), and ‐ ultimately ‐ of the charge (n=0 or 1) affected their *in vitro* anticancer properties. Cytotoxicity studies performed on two cancerous cell lines and one non‐tumorigenic cell line in cellular monolayer cultures revealed that the DIP complexes **3**–**5** displayed much higher bioactivities than the corresponding phen complexes **6**–**8**. In addition, the cationic acac complex [Ru(DIP)_2_(η^2^‐acac)]Cl (**4**) was found to be remarkably more cytotoxic compared to the neutral ones, with IC_50_ values in the mid‐nanomolar range and comparable to those of **1** and of cisplatin. Compound **4** also showed an excellent uptake in CT‐26 cancer cells, at least three times larger than the neutral DIP complexes of similar lipophilicity **3** and **5**, and inhibited their mitochondrial respiration. Conversely, the uptake of the neutral complexes was lower and they did not significantly affect mitochondrial respiration, thus suggesting also a different intracellular distribution (and, possibly, cytotoxic mechanism).

These results indicate that cationic polypyridyl‐dioxo Ru(II) complexes perform much better than neutral ones and that diimine ligands with large lipophilicity (DIP vs. phen) are to be preferred. Whereas the effect of the lipophilicity of the diimine ligand was somehow expected, the effect of the charge was not obvious, even though consistent with previous results from our and other groups. In a series of complexes in which different catecholate‐type dioxo ligands were bound to the {Ru(DIP)_2_} core, we found that cationic complexes with semiquinonate ligands were much more cytotoxic compared to neutral catecholate compounds.[^33^, ^34^, ^35^] Consistently, the mono‐cationic cyclometalated Ru(II) complex [Ru(bpy)(phpy)(dppz)]^+^ was found to be rapidly taken up by cancer cells, and nearly 90 % of the complex accumulated in their nuclei after a 2 h incubation.^[37]^ On the other hand, a 2+ charge ‐ even in the presence of good lipophilicity ‐ does not seem to be appropriate for anticancer activity: Barton et al. reported that, in the dppz‐based Ru(II) complex [Ru(DIP)_2_(dppz)]^2+^, the lipophilic DIP ligand facilitates complex cellular uptake into the cell cytoplasm but has low inhibition against different tumour cells as it cannot enter into the cell nucleus.^[38]^ In a recent paper, Glazer et al. investigated a series of cationic [Ru(bpy)_2_(O^O)]^+^ complexes, where O^O is acac or a substituted acac.^[24]^ They found that the *in vitro* cytotoxicity of the complexes roughly correlates with the lipophilicity/hydrophobicity of the acac ligand (and thus of the complex).^[24]^ The cytotoxic activity of complex **4** is consistent with these results; in Glazer's complexes, the lipophilicity of the substituted‐acac ligands compensates for the less lipophilic bpy ligand (compared to DIP).

Taken together, these results suggest that, for being effective, Ru(II) polypyridyl complexes not only must enter the interior of cells (i. e., have good lipophilicity) but must also be able to reach some important cellular organelles like the cell nucleus and/or the mitochondria (i. e., have a mono‐positive charge). In agreement with Glazer's findings on the good stability of the [Ru(bpy)_2_(O^O)]^+^ complexes, also in our case we might say that ligand exchange is not a key component of their mechanism of action.

Even though complex **4** showed no selectivity towards the CT‐26 and PC‐3 cancer cell lines compared to the healthy cell line RPE‐1, in the future, it might be worth to extend such *in vitro* investigation towards a broader panel of cell lines for ascertaining if this is a general feature. In addition, the problem of low selectivity could be overcome by encapsulation or by conjugation of the complex to targeting biomolecules (e. g., peptides or antibodies).[^39^, ^40^]

Finally, with these SAR results at hand, and considering the complexes described in this work, we anticipate that complex **4** – with its promising anticancer properties and excellent cell uptake – could be used as a model to synthesize novel Ru(II)‐based anticancer drugs. In particular, it might be worth investigating analogues of compound **4** with acac derivatives of increasing lipophilicity. It would also be helpful to assess the *in vivo* toxicity of such complexes against zebrafish to establish whether, like some of Glazer's compounds, they might have a favourable therapeutic window suitable for future *in vivo* studies.

## Experimental Section

**Materials**: All chemicals were obtained from commercial sources and were used without further purification. Compounds **3**–**8** were prepared and fully characterized as described in ref. 36. Spectroscopic data were in accordance with the literature.

**Stability in human plasma**: The stability of the complexes was evaluated upon incubation in pooled human plasma with caffeine as an internal standard, which was previously demonstrated to be stable under these conditions. Stock solutions of each complex (2 mM) and caffeine (2 mM) were prepared in dimethyl sulfoxide. 50 μL of both stock solutions (complex and caffeine) were added to 400 μL of human plasma, achieving 500 μL. The resulting solutions were incubated upon continuous gentle shaking (ca. 300 rpm) for 48 h at 37 °C. After this time, the incubation was ended by adding 1 mL of methanol. The mixture was centrifuged for 45 min at 3000 rpm and 4 °C. The solution was filtered through a 0.2 μm membrane filter and submitted to HPLC analysis, that were carried out on a Jupiter Proteo column (250×4.6 mm, 4 μm, flow rate: 1 mL/min). The solvents (HPLC grade) were Millipore water (solvent A) and acetonitrile (solvent B). Method M1 (compounds **3**–**5**): 0–3 min: isocratic 75 % A (25 % B); 3–30 min: linear gradient from 75 % A (25 % B) to 0 % A (100 % B) 30–35 min: isocratic 0 % A (100 % B); Method M2 (compounds **6**–**8**): 0–3 min: isocratic 95 % A (5 % B); 3–30 min: linear gradient from 95 % A (5 % B) to 0 % A (100 % B); 30–35 min: isocratic 0 % A (100 % B). The flow rate was 1 mL min^−1^, and the chromatogram was detected at 215 nm.

**Log P value determination**: Octanol and PBS were saturated with each other by continuous mixing at room temperature for 24 h. The test compounds (DMSO stock solutions) were added at a concentration of ca. 200 μM in 1 mL of the octanol phase. An equal volume of the PBS phase was added, and the mixture was agitated at room temperature for 24 h. The two layers were separated, and the absorbance of each layer was determined at 450 nm. The log P values were determined as follows:

$$\log P=log\left(\frac{{Abs}_{450}\left(Octanol\right)}{{Abs}_{450}\left(PBS\right)}\right)$$

**Cell cultures**: The CT‐26 cell line was cultured in DMEM media (Gibco) supplemented with 10 % foetal calf serum (Gibco) and 1 % penicillin‐streptomycin antibiotic (Gibco). The PC‐3 cell line was cultured in F12‐K media (Gibco) supplemented with 10 % foetal calf serum (Gibco) and 1 % Penicillin‐Streptomycin antibiotic (Gibco). The RPE‐1 cell line was cultured in DMEM/F‐12 media (Gibco) supplemented with 10 % foetal calf serum and 1 % Penicillin‐Streptomycin antibiotic (Gibco). Cell lines were maintained in a humidified atmosphere at 37 °C with 5 % CO_2_.

**Cytotoxicity assay using a 2D cellular model**: The cytotoxicity of the tested Ru complexes was assessed by a fluorometric cell viability assay using Resazurin (Acros Organics). Briefly, cells were seeded in triplicate in 96‐well plates at a 4×10^3^ cells/ well density in 100 μL. After 24 h, cells were treated with increasing concentrations of the ruthenium complexes and controls. Dilutions for complexes **3**–**8** were prepared as follows: 10 mM stock solution in DMSO was diluted to 100–0.01 μM with medium. After 48 h of incubation, the medium was removed, and 100 μL of complete medium containing resazurin (0.2 mg/mL final concentration) was added. After 4 h of incubation at 37 °C, the fluorescence signal of the resorufin product was read (ex 540 nm, em 590 nm) in an Infinite 200 PRO Microplate Reader from TECAN. IC50 values were then calculated using GraphPad Prism software.

**ICP‐MS cellular uptake studies**: CT‐26 cells were seeded at a density of 1×10^6^. The next day, cells were treated with 5 μM of the corresponding complex diluted in the cell culture medium from a 10 mM stock solution in DMSO. After 2 h, cells were collected, counted, and stored at −80 °C. ICP‐MS samples were prepared as follows: samples were digested using 70 % nitric acid (1 mL, 60 °C, overnight) and then further diluted 1 : 100 (1 % HCl solution in MQ water) analysed using ICP‐MS. All ICP‐MS measurements were performed on an Agilent 7900 Quadrupole ICP‐MS located at the Institut de Physique du Globe de Paris (France). The monitored isotopes are 99 and 101 Ru. Daily, before the analytical sequence, an indium internal‐standard was injected after inline mixing with the samples to correct for signal drift and matrix effects. A set of calibration standards was analysed to confirm and model (through simple linear regression) the linear relationship between signal and concentration. The model was then used to convert measured sample counts to concentrations. Reported uncertainties were calculated using error propagation equations and considering the combination of standard deviation on replicated consecutive signal acquisitions (n=3), internal‐standard ratio and blank subtraction. The non‐linear term (internal‐standard ratio) was linearized using a first‐order Taylor series expansion to simplify error propagation. The amount of metal detected in the cell samples was transformed from ppb to μg of metal. Data were subsequently normalized to the number of cells and expressed as μmol of metal/number of cells.

*Sample preparation for cellular fractionation*: CT‐26 cells were seeded in three 15 cm^2^ cell culture dishes and cells were 90 % confluent on the day of treatment. On the day of treatment, cells were incubated with the target complex at a concentration of 5 μM for 4 h, then the medium was removed and the cells were washed, collected, and counted. After re‐suspension in cold PBS, the organelles were isolated by using different protocols (one cell culture dish per isolation was used).

*Mitochondria and nucleus isolation* : To isolate mitochondria, a Mitochondria Isolation Kit (Cat. Nr: MITOISO2, SigmaAldrich) and Nucleus Isolation Kit (*NUC‐101)* were used according to the manufacturer procedure for the isolation of mitochondria via homogenization method.

**Metabolic studies: mito stress test**: CT‐26 cells were seeded in Seahorse XFe 96‐ well plates at a density of 20 000 cells/well in 80 μL of the medium. After 24 h, the medium was replaced with fresh medium, and either complex **1** (1 μM), **3** (5 μM), **4** (0.5 μM), **5** (10 μM), **6** (25 μM), **7** (10 μM), or **8** (25 μM) was added. After 24 h of incubation, the regular medium was removed, and cells were washed three times using Seahorse base media and incubated in a non‐CO_2_ incubator at 37 °C for 1 h. A Mito stress assay was run using 1 μM oligomycin, 1 μM FCCP, and a mixture of 1 μM antimycin‐A/1 μM rotenone in ports A−C, respectively, using a Seahorse XFe96 extracellular flux analyser.

## Conflict of interest

The authors declare no conflict of interest.

1

## Supporting information

As a service to our authors and readers, this journal provides supporting information supplied by the authors. Such materials are peer reviewed and may be re‐organized for online delivery, but are not copy‐edited or typeset. Technical support issues arising from supporting information (other than missing files) should be addressed to the authors.

Supporting Information

## Data Availability

The data that support the findings of this study are available in the supplementary material of this article.
