# Supplementary material for: Ruthenium(II) Polypyridyl Complexes Containing Simple Dioxo Ligands: a Structure‐Activity Relationship Study Shows the Importance of the Charge
Source: Chembiochem. 2022 Aug 26;23(19):e202200398. doi: 10.1002/cbic.202200398 (PMC9804910; doi:10.1002/cbic.202200398)

ChemBioChem

Supporting Information

Ruthenium(II) Polypyridyl Complexes Containing Simple Dioxo Ligands: a Structure-Activity Relationship Study Shows the Importance of the Charge

Albert Gandioso, Alessio Vidal, Pierre Burckel, Gilles Gasser,* and Enzo Alessio*

INDEX

1. **Figure S1.** Time-dependent UV-Vis spectra of complexes **3** – **8** (1 mM) in DMSO.
2. **Figure S2.** Time-dependent UV-Vis spectra of complexes **3** – **8** (1 mM) in DMEM cell culture medium.
3. **Figure S3.** Time-dependent UV-Vis spectra of complexes **3** – **8** (1 mM) in Human Plasma.
4. **Figure S4.** Reversed-phase HPLC traces at 215nm for the stability experiments in Human Plasma of complexes **3** – **5**.
5. **Figure S5.** Reversed-phase HPLC traces at 215nm for the stability experiments in Human Plasma of complexes **6** – **8**.
6. **Figure S6.** Fluorometric cell viability assay of compounds **3** – **5** and **7** in CT26 cell line.
7. **Figure S7.** Fluorometric cell viability assay of compounds **3** – **5** (plus **1** and cisplatin) in PC3 cell line.
8. **Figure S8.** Fluorometric cell viability assay **3** – **5** (plus **1** and cisplatin) in RPE-1 cell line.

Figure S1. Time-dependent UV-Vis spectra of complexes **3** – **8** (1 mM) in DMSO.

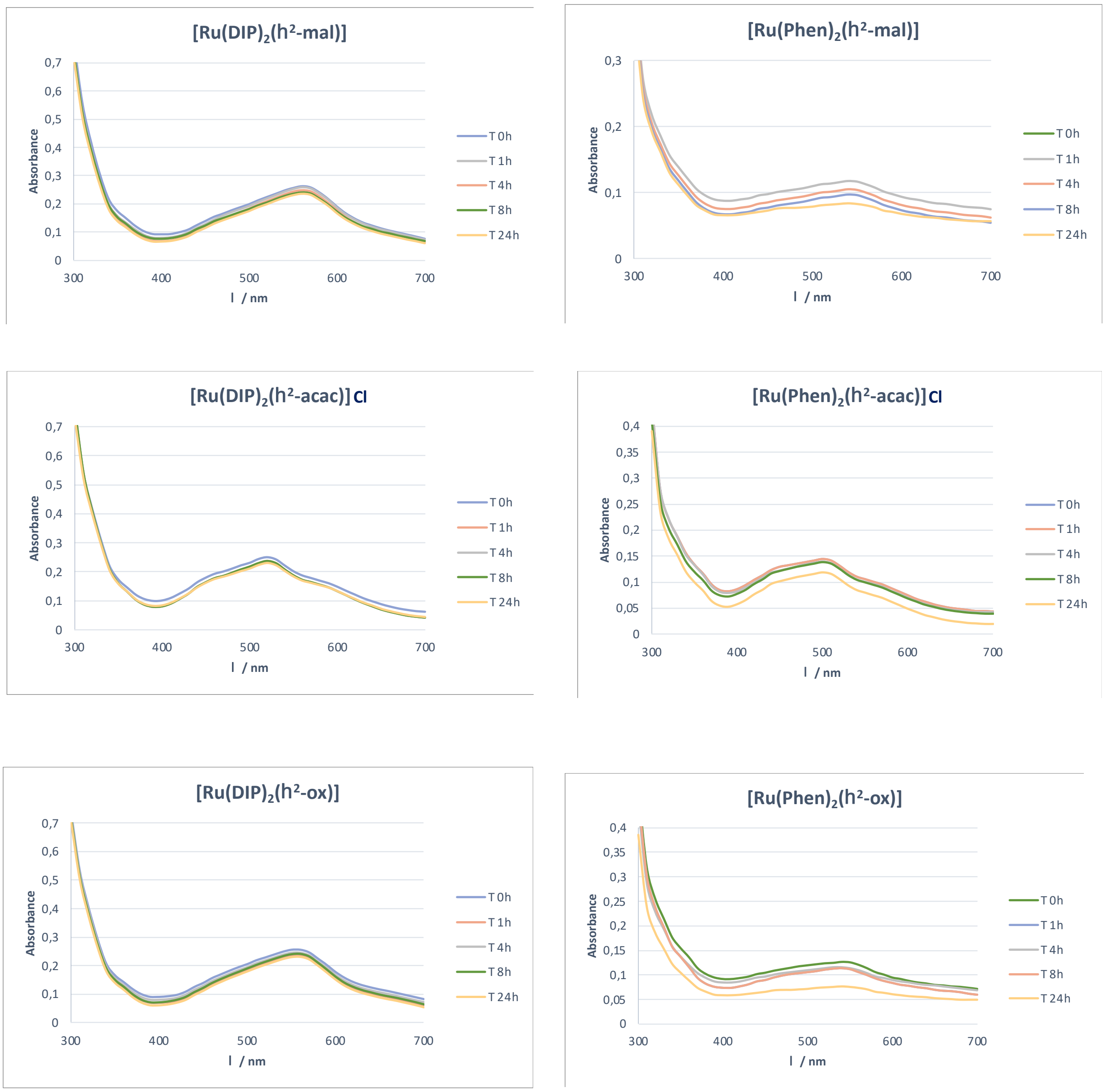

Figure S2. Time-dependent UV-Vis spectra of complexes **3** – **8** (1 mM) in DMEM cell culture medium.

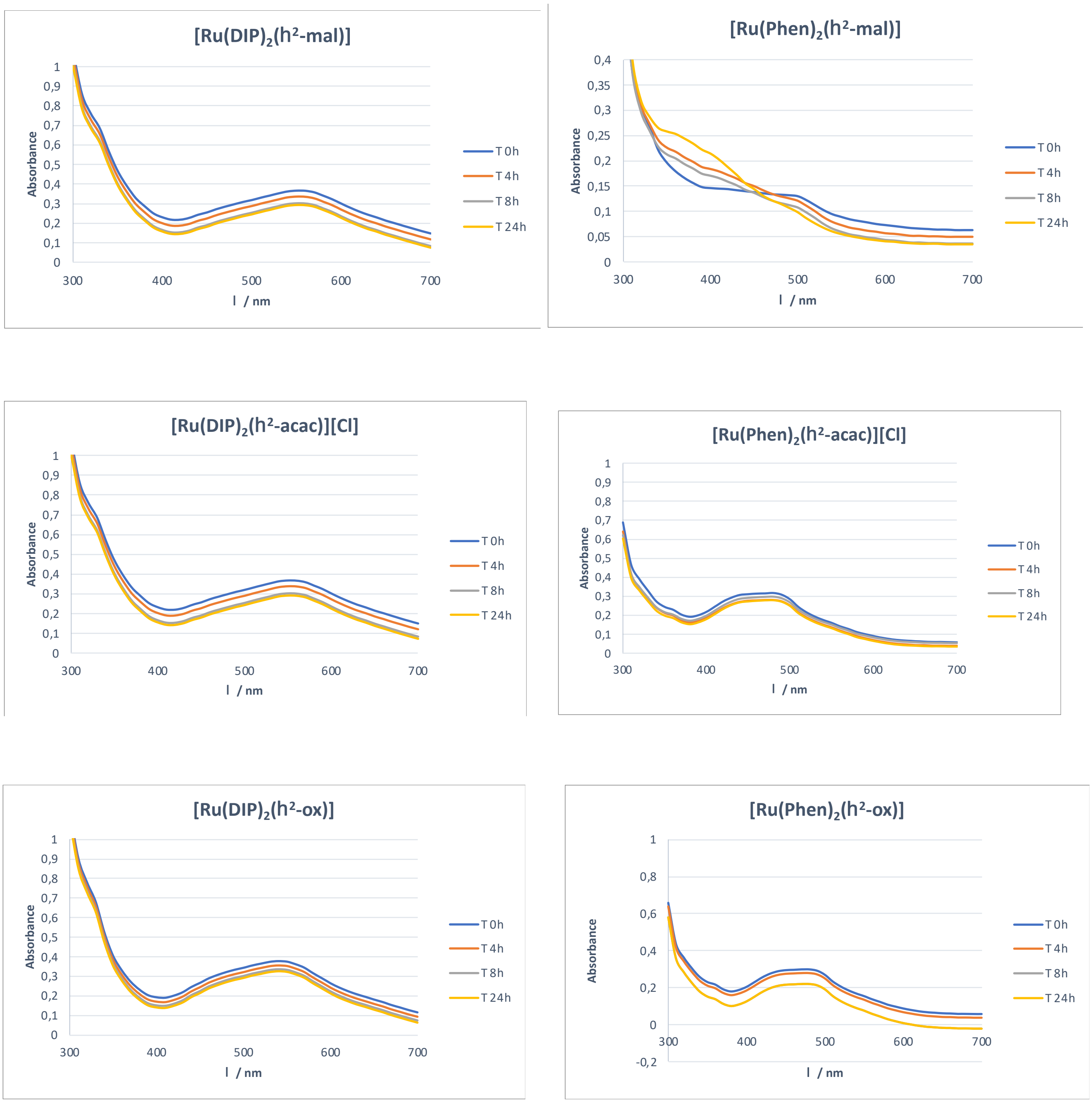

Figure S3. Time-dependent UV-Vis spectra of complexes **3** – **8** (1 mM) in Human Plasma.

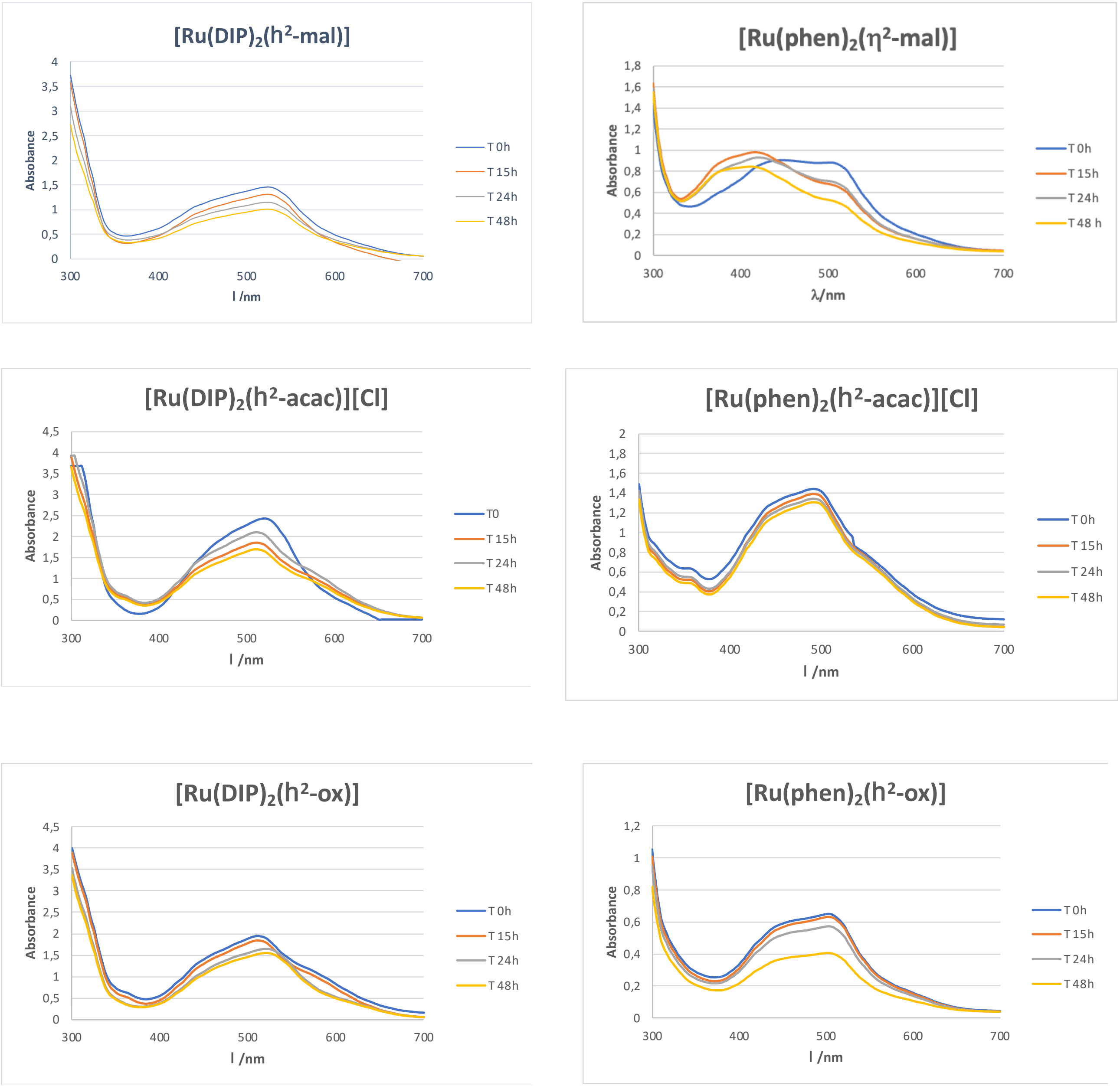

Figure S4. Reversed-phase HPLC traces at 215nm for the stability experiments in Human Plasma of complexes **3** – **5** in the presence of Caffeine (internal standard). Gradient: 25% ACN in H_2O to 100% ACN in 30 min.

[Ru(DIP)$_2$(η^2-mal)] (**3**) Caffeine (Rt = 4 min) , **3** (Rt = 30.96 min),

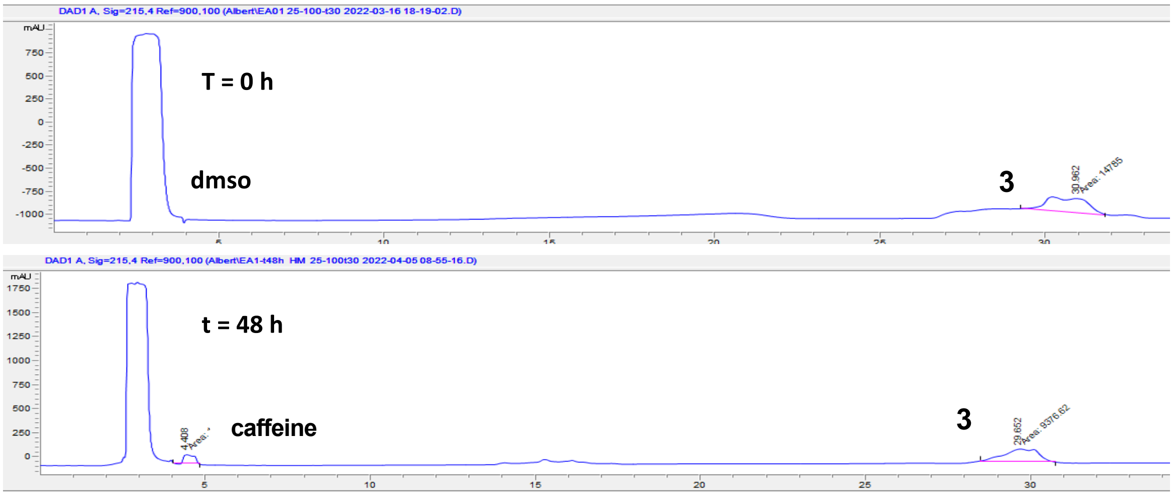

[Ru(DIP)$_2$(η^2-acac)][Cl] (**4**) Caffeine (Rt = 4 min) , **4** (Rt = 20.20 min)

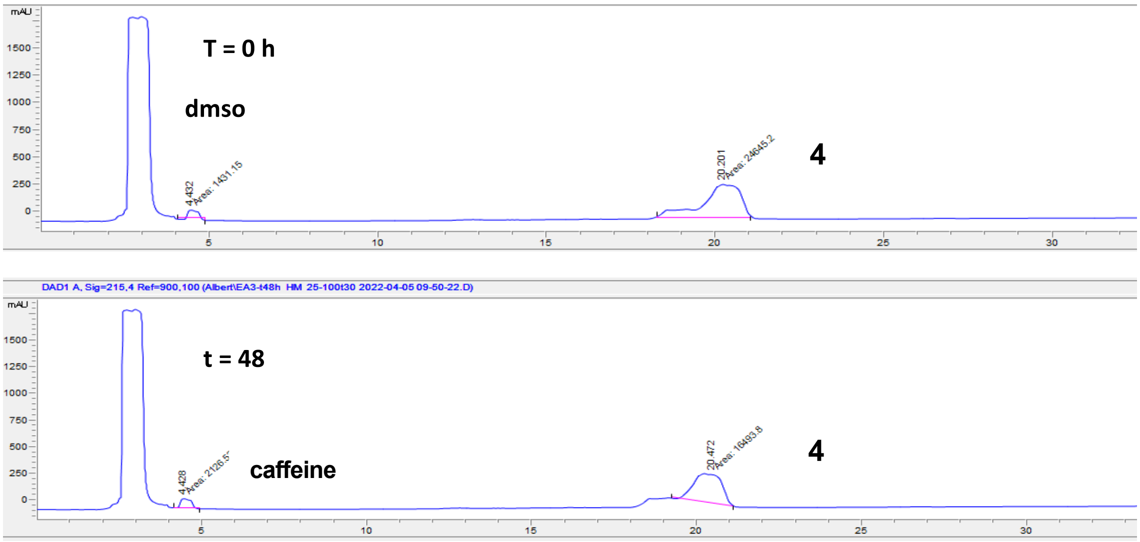

[Ru(DIP)$_2$(η^2-ox)] (**5**) Caffeine (Rt = 4 min) , **5** (Rt = 29.10 min)

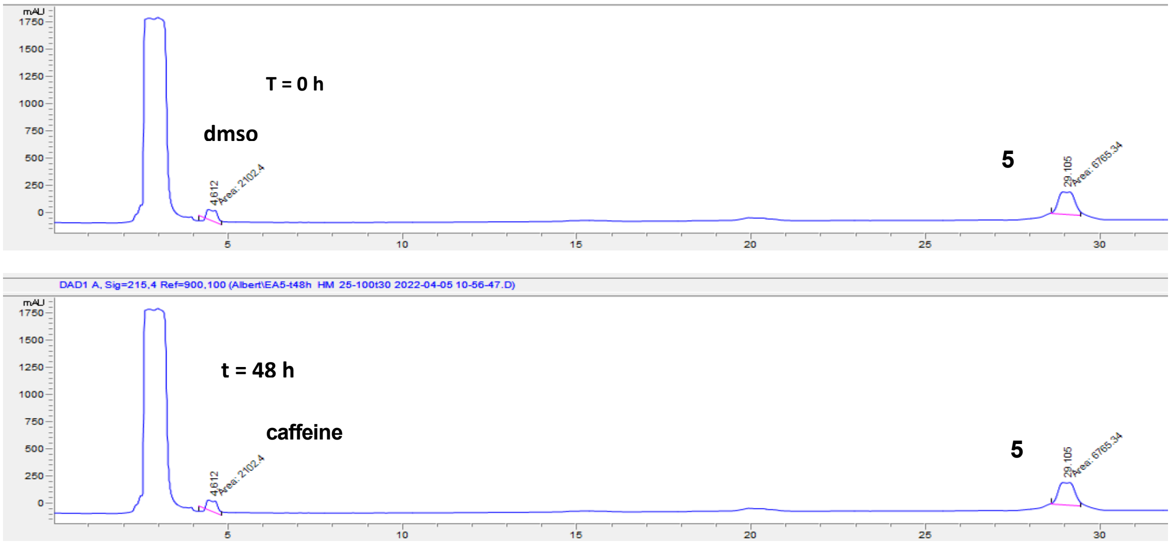

Figure S5. Reversed-phase HPLC traces at 215nm for the stability experiments in Human Plasma of complexes **6** – **8** in the presence of caffeine (internal standard). Gradient: 5% ACN in H_2O to 100% ACN in 30 min.

[Ru(phen)$_2$(η^2-mal)] (**6**) *injection peak ; Caffeine (Rt = 12.4 min) , **6** (Rt = 14.64 min), **6b** (Rt =12.1 min)

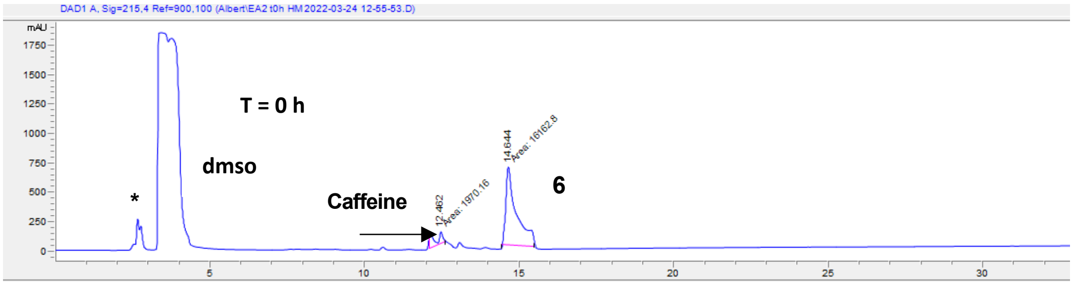

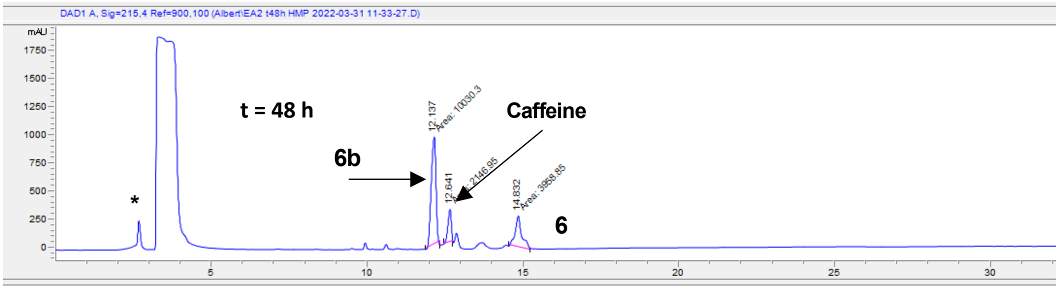

[Ru(phxen)$_2$(η^2-acac)][Cl] (**7**) : *injection peak ; Caffeine (Rt = 12.17 min) , **7** (Rt = 14.02 min)

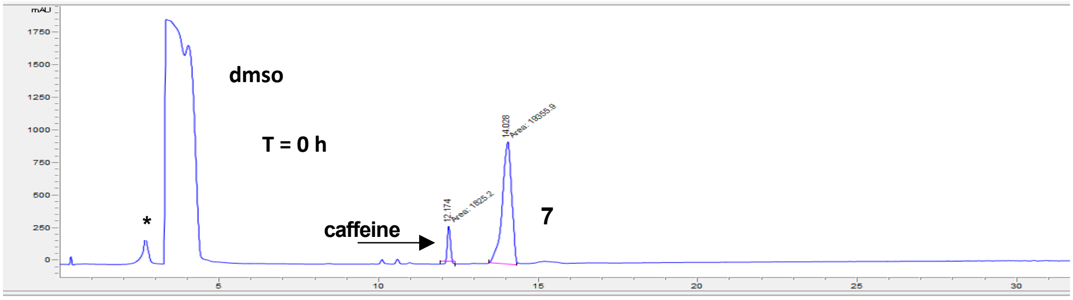

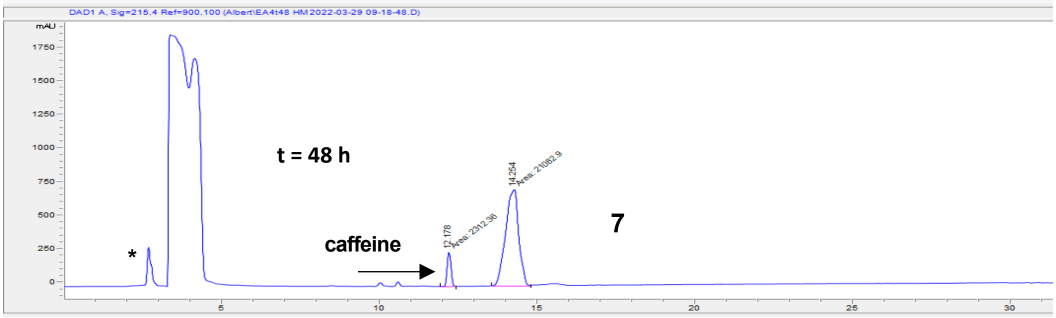

[Ru(phen)$_2$(η^2-ox)] (**8**) : *injection peak ; ^ intrinsic of **8** , Caffeine (Rt = 12.18 min) , **8** (Rt = 15.08 min)

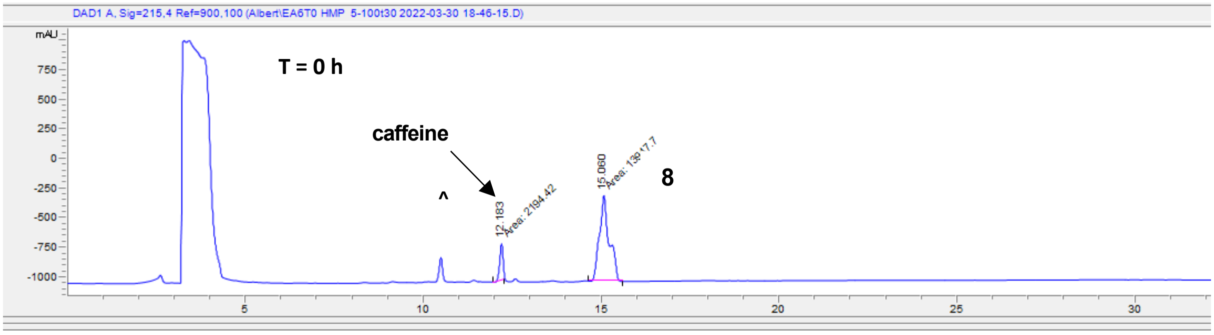

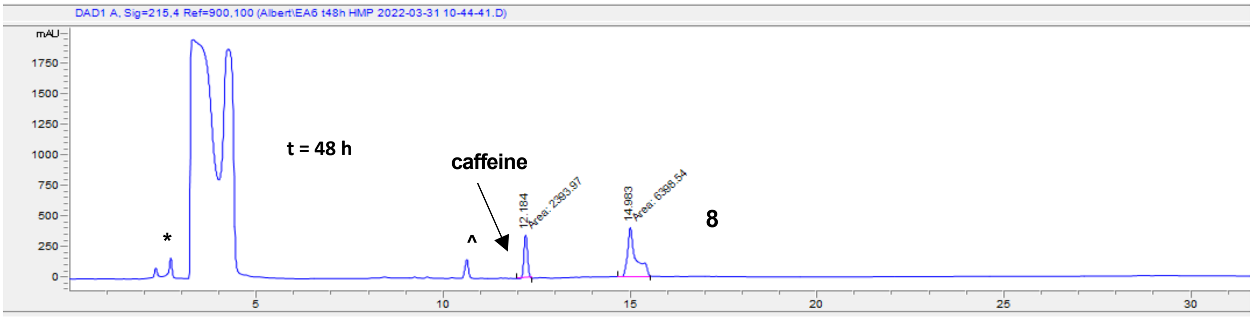

Figure S6. Fluorometric cell viability assay of compounds **3** – **5** and **7** in CT26 cell line.

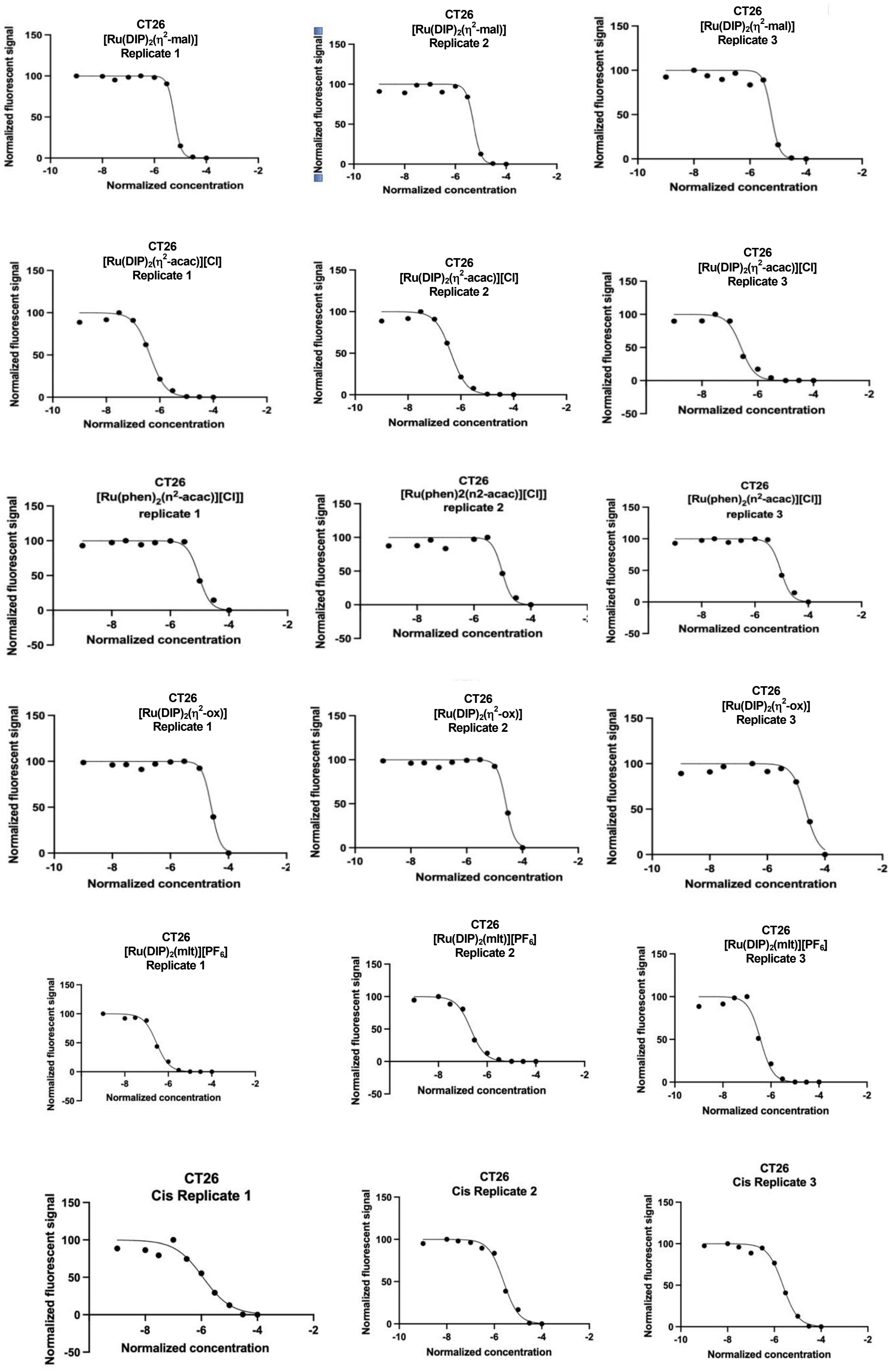

Figure S7. Fluorometric cell viability assay of compounds **3** – **5** (plus **1** and cisplatin) in PC3 cell line.

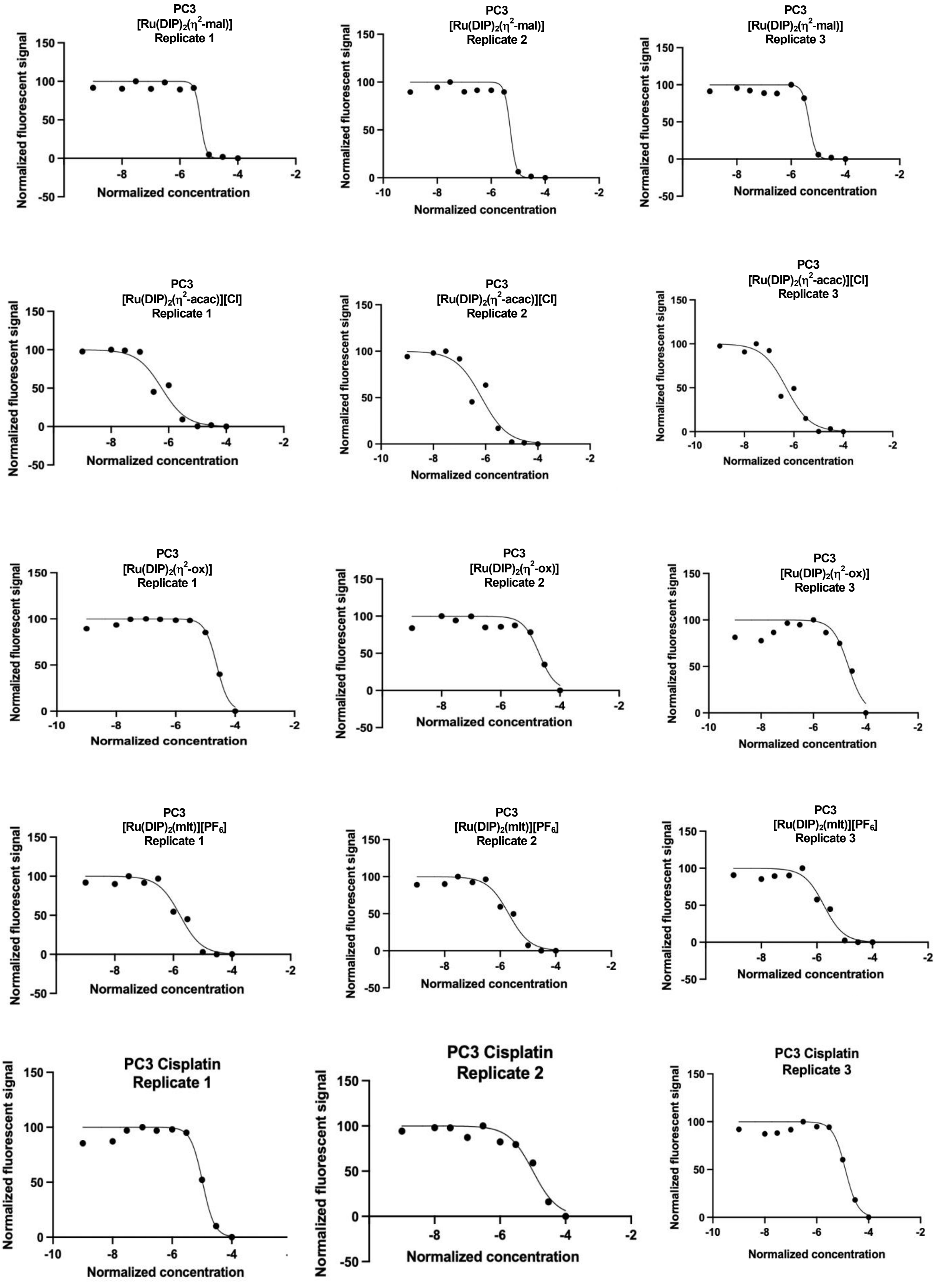

Figure S8. Fluorometric cell viability assay **3** – **5** (plus **1** and cisplatin) in RPE-1 cell line.

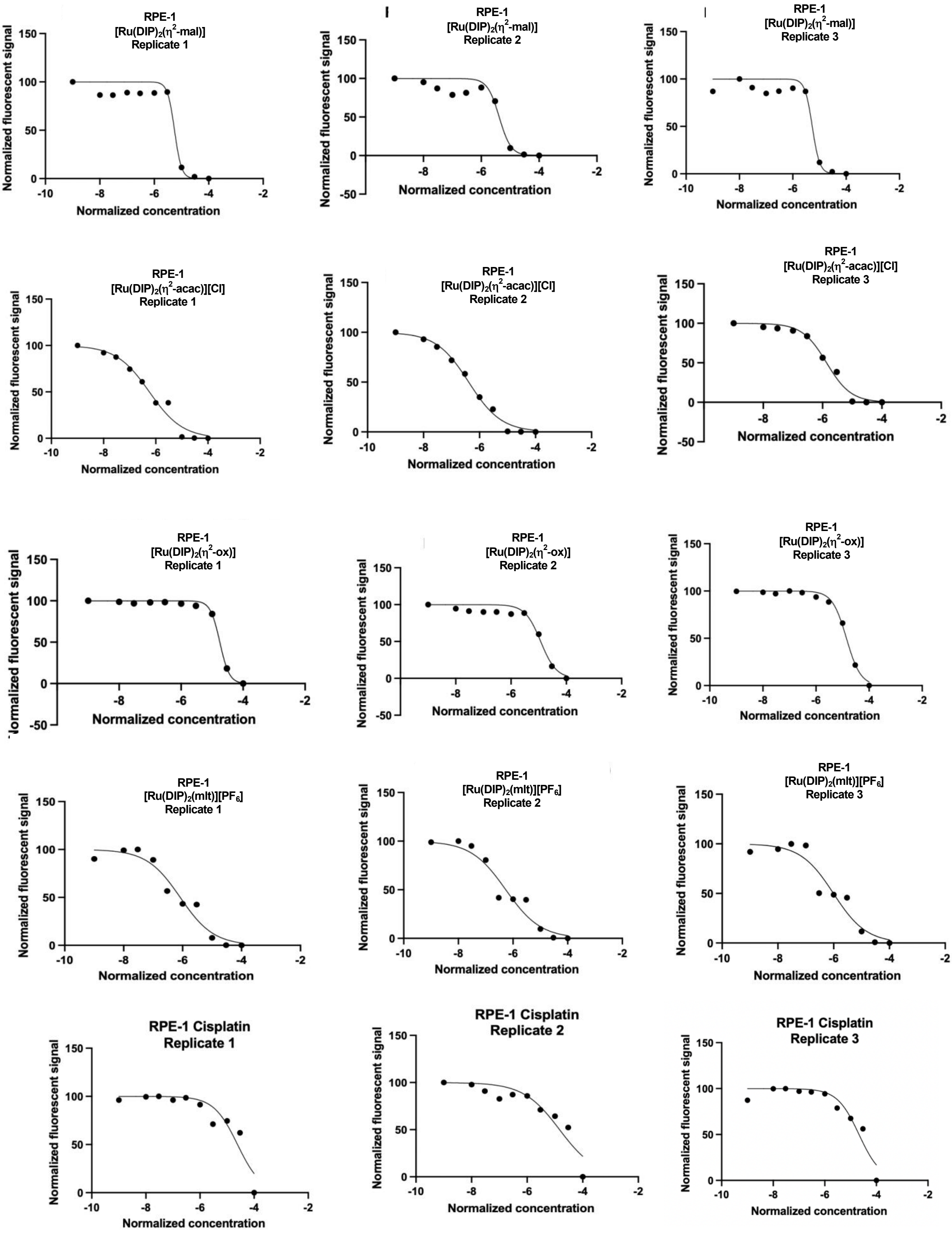

Supplement: Supplementary file 1 — Supporting Information [file CBIC-23-0-s001.pdf]
